# The evolution of nutritional care in children with food allergies – With a focus on cow's milk allergy

**DOI:** 10.1111/jhn.13391

**Published:** 2024-11-25

**Authors:** Rosan Meyer, Marion Groetch, Alexandra Santos, Carina Venter

**Affiliations:** ^1^ Department of Nutrition and Dietetics University of Winchester Winchester UK; ^2^ Department of Medicine KU Leuven Leuven Belgium; ^3^ Department of Pediatrics, Icahn School of Medicine at Mount Sinai Division of Pediatric Allergy and Immunology New York New York USA; ^4^ Department of Women and Children's Health (Pediatric Allergy), School of Life Course Sciences, King's College London Faculty of Life Sciences and Medicine London UK; ^5^ Peter Gorer Department of Immunobiology, School of Immunology and Microbial Sciences King's College London London UK; ^6^ Children's Allergy Service, Evelina London Children's Hospital Guy's and St Thomas’ Hospital London UK; ^7^ Section of Allergy and Immunology University of Colorado/Children's Hospital Colorado Boulder Colorado USA

**Keywords:** children, cow's milk allergy, dietary management, evolution, food allergies, nutrition

## Abstract

Cow's milk allergy (CMA) remains one of the most common and complex paediatric food allergies. In the last decade, our understanding has advanced in terms of immunoglobulin E (IgE)‐mediated CMA and focus is now also paid to non‐IgE‐mediated CMA, particularly in some Western countries where incidence rates are high. We have had significant progress in the last 10 years in relation to our understanding of existing supportive tests for IgE‐mediated CMA, with the advancement of newer tests, such as the basophil activation test (BAT), which have shown great promise. However, little advancement has been made in terms of tests for non‐IgE‐mediated CMA, and controversy still exists around symptoms. Our understanding of the natural history of CMA has also advanced with more awareness of different phenotypes. While the mainstay of management remains cow's milk elimination, the importance of supporting breastfeeding and avoidance of unwarranted cow's milk elimination diets in breastfeeding mothers has been highlighted. For non‐breastfed children, there has been some advancement in the formulas offered for the management of CMA, including the recognition of hydrolysed rice‐based formulas and increased demand for nutritionally complete plant‐based options, some of which are currently being assessed. The addition of pro, pre and synbiotics is considered safe to use, although research and guidance on routine use remain absent. Knowledge of tolerance induction from studies on the early introduction of peanuts has also highlighted the importance of a more active approach to managing CMA with the use of milk ladders, primarily in non‐IgE‐mediated CMA and baked milk (BM) introduction in IgE‐mediated CMA. In addition, modulation of the microbiome and diet diversity during complementary feeding has been a major advancement in the last 10 years. While data on poor growth and feeding difficulties in children with CMA has not changed much, increased rates of obesity are now also reported. Finally, novel approaches, including oral immunotherapy, the use of milk ladders and earlier consideration of BM, have advanced somewhat in the last decade, although the risks and benefits of these novel approaches require further research. While CMA remains a complicated allergy to diagnose and manage, the evolution of science has advanced our knowledge and brought some novel innovations, which combined have enhanced our practice.

## Key points

- We have had significant progress in the last 10 years in relation to our understanding of existing supportive tests for immunoglobulin E‐mediated cow's milk allergy such as the basophil activation test.
- Supporting breastfeeding and the unwarranted cow's milk elimination diets in breastfeeding mothers has been highlighted as a concern.
- There has been some advancement in the formulas offered for non‐breastfed infants, which include the increased recognition of hydrolysed rice‐based formulas and demand for nutritionally complete plant‐based options.
- Novel approaches including oral immunotherapy, the use of milk ladders and the earlier consideration of baked milk have advanced in the last decade although the risks and benefits of these novel approaches require further research.

## Graphical abstract

Both over and underdiagnosis have nutritional consequences in children with cow's milk allergy. It is, therefore, important to have robust diagnostic algorithms in place. Supporting breastfeeding and recommending the appropriate cow's milk alternative is critical in the management to ensure optimal growth and development, with some advances being made with plant‐based alternatives. Active management, including the earlier introduction of baked milk and the use of a milk ladder in targeted populations, are recent advances.

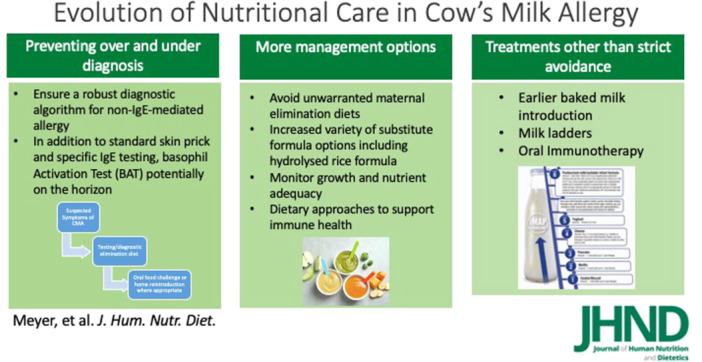

## INTRODUCTION

Cow's milk allergy (CMA) is one of the most common and complicated food allergies seen in children worldwide. In the past, overall prevalence data have primarily focused on immunoglobulin E (IgE) mediated CMA, which ranged from 1.8% to 7.5% between 1973 and 2008.^1^ In 2015, the EuroPrevall birth cohort based on 12,049 children, published the incidence of CMA across Europe, which included both IgE and non‐IgE‐mediated allergy.^2^ The overall challenge‐proven incidence was reported to be 0.54%, but there was a large variation seen in the incidence of non‐IgE‐mediated CMA. For example, in the United Kingdom, this study reported more children as having non‐IgE‐mediated CMA (56.3%) than IgE‐mediated CMA (43.7%), while in other countries such as Spain, Lithuania, Greece and Germany, there was no reported incidence of non‐IgE‐mediated CMA.^2^ This was highlighted as one of the limitations of the study, indicating the screening procedure may have lacked sensitivity for non‐IgE‐mediated gastrointestinal manifestations of CMA.^3^

While the presentation and recognition of IgE‐mediated CMA symptoms have not changed over the years, including hives, angio‐oedema, atopic dermatitis, facial/lip swelling, and in the severest of cases cardio‐respiratory symptoms, there has been an increased recognition of non‐IgE‐mediated CMA.^4^ Non‐IgE‐mediated CMA symptoms are typically delayed, affecting the gastrointestinal tract (i.e., vomiting, diarrhoea, constipation, abdominal pain) and skin (i.e., atopic dermatitis). However, the most notable advancement has been the recognition that cow's milk protein is the primary culprit allergen in the development of food protein‐induced enterocolitis syndrome (FPIES) in infants with a reported prevalence of 0.34%.^5^ While acute FPIES is easier to recognise due to repeated profuse vomiting after exposure, other non‐IgE‐mediated CMA symptoms commonly overlap with disorders of paediatric gut–brain interaction (previously called functional paediatric disorders), and there is also a lack of an accurate diagnostic test.^6^ Experts have therefore cautioned against overdiagnosing non‐IgE‐mediated CMA,^7^ which is compounded by the fact that many parents believe their child to be food allergic, while food challenge‐based data is significantly lower.^8^

CMA typically presents in early childhood following exposure to cow's milk‐based infant formula, or with the introduction of cow's milk containing complementary foods.^9^ Meanwhile infants exhibit the highest growth velocity around this age with other critical developmental milestones (e.g., ability to eat solid foods), emphasising the importance of early recognition and optimal management of CMA. There has been significant advancement in both the diagnosis and management of CMA, with an improved understanding of the disease pathology, which will be discussed in this review.

## DIAGNOSIS OF CMA

It is well established that the diagnosis of CMA is dependent on a detailed allergy‐focused history.^10^ The symptoms and timing of presentation of IgE‐mediated allergy (Table 1) have been recognised for at least 20 years. However, controversy still exists around some of the symptoms associated with non‐IgE‐mediated allergy, including anal excoriation, anal fissures and irritability.^11^

**Table 1:** Typical symptoms for IgE and non‐IgE‐mediated CMA.^4^, ^11^, ^12^

IgE‐mediated CMA	Non‐IgE‐mediated CMA
**Presents** – within minutes up to 1 h after exposure to milk formula or dairy products **Cutaneous** – urticaria, angioedema, eczema **Digestive** – vomiting, diarrhoea **Respiratory** – rhinitis, cough, wheezing, stridor, dyspnoea **Cardiovascular or neurological** – pallor, dizziness, floppiness, hypotension, loss of consciousness	**Presents** – >2 h and up to 3 days after exposure, but for FPIES symptoms can present 1–4 h after allergen exposure **Cutaneous** – eczema **Digestive** – profuse/chronic vomiting, diarrhoea, abdominal pain/discomfort, constipation **General** – faltering growth, feeding difficulties and with eosinophilic oesophagitis (EoE) food impaction

Abbreviations: CMA, cow's milk allergy; IgE, immunoglobulin E.

For IgE‐mediated CMA there is the possibility to assess IgE sensitisation using supportive tests in association with a positive history to aid diagnosis and also prognosis. Studies comparing the results of these supportive tests, for example, skin prick tests (SPT) and specific IgE tests with an oral food challenge (OFC) to foods, have allowed predictive values to be developed which guide the interpretation in individual patients.^12^ A recent systematic review by the European Academy of Allergy and Clinical Immunology (EAACI) gives useful guidance on the interpretation of specific IgE and SPT results and cut‐off levels (Table 2). However, it is important to consider that these cut‐off levels may be population‐specific. Such cut‐offs should be interpreted taking demographic and clinical information about the specific patient into consideration, as well as the prevalence and severity spectrum of the food allergy in the specific patient population.^13^

**Table 2:** Diagnostic performance of various tests for allergy to fresh pasteurised cow's milk based on a recent meta‐analysis of diagnostic studies for all ages.^13^

Diagnostic tests	Cut‐offs	Sensitivity	Specificity
	(95% CI)	(95% CI)	(95% CI)
Skin prick test to cow's milk extract (mm)	4 (3–8)	0.52 (0.24–0.79)	0.8 (0.65–0.90)
Specific IgE to cow's milk extract (KU/L)	3.5 (0.9–10.5)	0.82 (0.59–0.94)	0.92 (0.80–0.97)
Specific IgE to casein (KU/L)	2.6 (1.0–5.3)	0.67 (0.53–0.78)	0.93 (0.85–0.97)

Abbreviation: IgE, immunoglobulin E.

Targeted skin prick or specific IgE testing is also important in non‐IgE‐mediated food allergy in particular if they have symptoms that may be immediate or eczema, to rule out IgE sensitisation and to confirm the non‐IgE‐mediated nature of symptoms. It is important to consider these in the context of the clinical history to avoid overdiagnosing IgE‐mediated allergies. However, the diagnosis in most non‐IgE‐mediated conditions (outside of eosinophilic oesophagitis, which requires an endoscopic confirmation and acute FPIES, where the diagnosis is based on the presentation of symptoms) is reliant on the elimination of cow's milk and in some cases other foods, followed by a period of reintroduction of cow's milk in the diet, to assess symptom recurrence. There has been some debate around the length of time needed on the elimination diet followed by the reintroduction, which in studies ranged from 2 to 8 weeks, but 4 weeks was considered sufficient, based on recent data, for the vast majority of patients with suspected non‐IgE‐mediated CMA.^14^, ^15^

In equivocal cases, for example, when IgE‐mediated food allergies or other severe symptoms are of concern, an OFC is required. If this controlled allergen exposure, under medical supervision, induces symptoms, food allergy is confirmed. On the other hand, if an age‐appropriate portion of the food is consumed without developing any symptoms, food allergy is excluded, and the suspected food can be included in the diet. New EAACI clinical guidelines on the diagnosis of IgE‐mediated food allergy have recently been published along with EAACI position papers on non‐IgE‐mediated food allergy, which provide excellent guidance for the diagnosis of these allergic conditions.^13^, ^15^, ^16^

Novel tests with higher accuracy than SPT and specific IgE testing have emerged to reduce the number of OFCs needed while improving the accuracy and safety of the food allergy diagnostic workup. The basophil activation test (BAT) is a functional test that uses flow cytometry to analyse the blood cells involved in allergic reactions, called basophils, and whether they degranulate following exposure to the allergen in vitro.^17^ Degranulation is measured by activation marker CD63. BAT was, for the first time, included in the EAACI Food Allergy Guidelines^13^; however, recommendations are limited to peanuts and sesame, as there is not enough evidence for use for the diagnosis of food allergies in other foods. BAT has been trialled for IgE‐mediated CMA with promising results, and studies on BAT to milk are ongoing (e.g., BAT2 study – NCT03309488). Component testing has been included in the EAACI Food Allergy Guidelines, for the first time, but cow's milk allergen components, like alpha‐lactalbumin, beta‐lactoglobulin and casein, do not seem to provide more information than IgE to cow's milk extract.^18^

In the future, it would be helpful if BAT was available to clinicians, ideally with more studies performed looking at other test modalities, such as IgE to allergenic peptides. It is especially important to look at in vitro tests to support the diagnosis of non‐IgE‐mediated CMA, for which biomarkers are highly needed to improve the objectivity and precision of the diagnostic process.

## NATURAL HISTORY OF CMA

**Figure 1:**
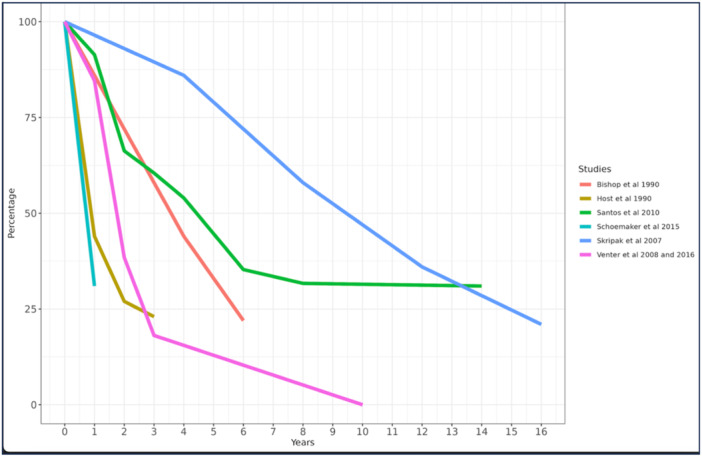
Comparison of natural history of cow's milk allergy over time.

CMA resolves spontaneously in most affected children, with a greater proportion of children with non‐IgE‐mediated CMA outgrowing this within the first year of life.^2^ Studies from the last decade have suggested a lower resolution rate in children with IgE‐mediated CMA. Natural history studies from the 90s reported resolution of CMA for about 87% of children by 3 years of age, whereas more recent studies suggest resolution only in about 60% of children by 6 years of age (Figure 1). These differences could be due, in part, to recent studies focusing more on patients seen in specialised centres rather than the general population and on IgE‐mediated rather than mixed IgE and non‐IgE‐mediated allergies. However, it could also be due to a change in phenotype and worsening prognosis, accompanying the increase in prevalence and severity of food allergies.^19^, ^20^

Factors contributing to the persistence of CMA over time include immediate‐type symptoms, respiratory symptoms, asthma, multiple food allergies, severe atopic eczema and large SPT wheal and high milk‐specific IgE levels at diagnosis.^20^, ^21^, ^22^ The skin reactivity on SPT and specific IgE to cow's milk can be re‐tested over time to determine symptom resolution or to determine the appropriate time for reintroductions of the food, often with repeated OFC. A decrease of >50% in specific IgE levels over 1 year or a decrease in levels below 50% PPV are encouraging signs, especially in children who have not had recent reactions to the allergens.^22^

While the prognosis of non‐IgE‐mediated CMA has not changed over time, based on recent studies, the allergic march (a medical term used to explain the natural history of atopic manifestations) for this delayed allergy is better understood (but not fully established).^23^ The most notable evolution in knowledge related to patients with a history of non‐IgE‐mediated CMA is that they seem to have a higher risk of developing paediatric disorders of gut–brain interaction.^24^

## EVOLUTION OF THE DIETARY MANAGEMENT OF CMA

### Breastmilk

In recent years, there has been significant debate around the existence of CMA in exclusively breastfed infants. This has been driven by several factors, including limited data on breastfeeding and CMA, varying levels of ß‐lactoglobulin in breastmilk, the clinical relevance of the presence of cow's milk protein in breastmilk and the more recent concern that companies manufacturing breastmilk substitutes for CMA have impacted negatively on breastfeeding rates.^25^ The most commonly quoted study on the presence of CMA in breastfed infants is by Høst et al.^26^ This study reported that 0.5% of the 2.2% of children diagnosed with challenge‐proven IgE‐mediated CMA, presented while being exclusively breastfed.^26^ Beyond this study, only observational and retrospective studies exist.

A recent review identified 27 studies assessing bovine milk protein in breast milk. These studies all included data on the type of sampling method, the sampling time, the lactation stage, maternal allergy status and most importantly the impact on the infant.^27^ They documented the presence of β‐lactoglobulin, a milk protein unique to cow's milk, in human breastmilk at similar levels previously published by Høst and Halken (range between 0.9 and −150 μg/L), with some breastfeeding mothers secreting no β‐lactoglobulin.^27^, ^28^ They reported that bovine protein was detected in samples up to 7–10 days after stopping the consumption of cow's milk,^27^ and there was a significant correlation between high levels of β‐lactoglobulin in breastmilk to clinical manifestations such as diarrhoea, vomiting, colic, or eczema, which are all typical non‐IgE‐mediated symptoms.^29^, ^30^ The theoretical possibility of these levels of β‐lactoglobulin leading to symptoms has been debated in a publication from 2020, based on the Eliciting Dose (ED) where 1% of children with IgE‐mediated CMA react. It was argued that only 1 in 600 breastmilk samples would contain sufficient β‐lactoglobulin to elicit a reaction and only 1% of the most sensitive patients with CMA.^31^ The probability therefore, according to this publication, of having enough β‐lactoglobulin in breastmilk to trigger an allergic reaction has been estimated to be as low as 1:2893.^27^

There are several problems with these theoretical calculations. The first and most obvious is that the ED01 is based on IgE‐mediated and not non‐IgE‐mediated CMA. Additionally, the testing method, presence of maternal atopy and the pathophysiology of non‐IgE‐mediated allergy have not been considered when calculating potential reactions to cow's milk protein from breastmilk. Studies have shown marked differences in human breastmilk between allergic and non‐allergic mothers, including the levels of short‐chain fatty acids,^32^ which impact the gut microbiota of the infant. Additionally, protease inhibitors and apolipoproteins were present in much higher concentrations in the breastmilk of allergic compared to non‐allergic mothers. These proteins have been suggested to be linked to both allergy and asthma.^33^ Levels of β‐lactoglobulin have also been found to be higher in the breast milk of allergic mothers.^34^ It has been hypothesised that maternal allergic status may impact the digestion and absorption of food due to increased gut permeability and therefore explain the higher levels of β‐lactoglobulin found in the milk of these mothers.^27^ Differences in the composition of breastmilk in atopic versus non‐atopic mothers have also been documented, which may also impact how infants respond.^30^, ^35^, ^36^ Finally, the authors of the recent scoping review have suggested that the quantitative evaluation of bovine β‐lactoglobulin in human milk by ELISA could give rise to misleading interpretations. The inconsistency of the results obtained with immunochemical methods has been demonstrated in previous studies when testing β‐lactoglobulin using ELISA which were not confirmed by high performance liquid chromatography or with tandem mass spectrometry.^37^ Further data is therefore required to understand the impact of bovine protein through breastmilk in cow's milk allergic infants.

Currently, all CMA guidelines suggest breastmilk as the first choice for infants, when available and mothers should be supported to continue.^11^, ^15^, ^38^, ^39^, ^40^ Additionally, it is also acknowledged that in the majority of cases, no maternal cow's milk elimination diet is required. However, there are cases where a maternal cow's milk elimination diet is warranted. In these cases, healthcare professionals need to be aware of the nutritional impact this can have on the breastfeeding mother and correct for any micronutrient deficits that arise; and most importantly, reintroduce cow's milk to confirm or refute the diagnosis after a 2–4 week elimination diet.^15^

### Formulas suitable for the management of CMA

For infants fed with formula either as a single source of nutrition or in combination with breastmilk/solid foods, a suitable hypoallergenic formula that has been tested and proven to be safe and well tolerated, and demonstrated growth in children with CMA, is recommended. Hypoallergenic formulas in the EU must comply with European Food Safety Authority (EFSA) regulations,^41^ in the United States to Food and Drug Administration (FDA) regulations and in the United Kingdom, the Food for Specific Groups (Food for Special Medical Purposes for Infants, Infant Formula and Follow‐on Formula) (Information and Compositional Requirements) Regulations 2020.^42^, ^43^ These formulas include non‐dairy (plant‐based) whole protein options, amino acid‐based/elemental or hydrolysed formulas. In recent years both extensive and partially hydrolysed rice‐based formulas have also been developed and include safety, growth and tolerance data.^44^, ^45^, ^46^ As a result, the Diagnosis and Rationale for Action against Cow's Milk Allergy (DRACMA) guidelines (2024) and the recent ESGPHAN guideline (2023) both suggest that when hydrolysed rice‐based formulas are available, they can be used for the dietary management of CMA.^11^, ^47^

In the past, soy formula was the primary plant‐based whole protein formula suitable for the management of CMA, but is not recommended as first line treatment of CMA in developed countries due to soya also being an allergen and frequently reported as a concomitant allergen also in children that have CMA.^11^, ^47^ However, with the increased demand for plant‐based alternatives, there are current feasibility and growth trials being performed on a new range of plant‐based formulas, for example, almond in CMA.

All current guidelines continue to advise against the use of other mammalian milk formulas or mammalian milk.^47^

### Commercially available plant‐based beverages

The availability of commercial plant‐based beverages has increased in recent years, but they are often not nutritionally adequate to support normal growth and development of infants and not suitable for children <1 year of age.^48^, ^49^, ^50^, ^51^ Commercial plant‐based beverages include soy, coconut, almond, rice, oat, hazelnut, cashew, walnut, pea, sesame, hemp, tigernut and quinoa, but their availability and nutritional composition differ worldwide. Important nutritional factors that should be considered when selecting plant‐based beverages include protein, fat (especially in children under 2 years) energy, calcium, vitamin B12, Vitamin D and iodine levels, as these beverages are not nutritionally complete.

The advice in current guidelines is that commercially available plant‐based beverages should ideally only be used in children from 2 years of age. However, if a child is eating well as established during a dietetic assessment, these plant‐based beverages could be used successfully as part of a nutritionally sound diet (Box 1) after one year of age.^52^

Box 1: **Factors to consider that may indicate a toddler is ready to transition to a commercial plant‐based beverage.**

- Is at least one year of age
- Eats a varied solid food diet with a variety of foods from each food group
- Gets at least 2/3 of their energy from the varied solid food diet
- Consumes no more than 16 ounces/500 mLs of milk substitute per day (this includes breastmilk, formula, and other dairy substitutes like yogurt)
- Eats age‐appropriate textures
- Gets enough protein and fat and micronutrients in the diet from the solid foods and the available milk substitute
- Has no feeding difficulties that may reduce food variety
- Has no known micronutrient deficiencies; and
- Has no religious/cultural dietary requirements that reduce the variety of foods consumed

### Additions of pre/pro and synbiotics in formulas for CMA

Great advances have been made to better understand the role of the microbiome in the development and tolerance induction of CMA.^53^ The addition of specific probiotic strains, such as *Lactobacillus rhamnosus* GG, was found to enhance the acquisition of oral tolerance more rapidly in infants with CMA and reduce atopic manifestations in later infancy in trials using comparison feeds without this probiotic strain.^54^, ^55^ The addition of synbiotics did not have an impact on tolerance acquisition but they have been shown to positively modify the gut microbiome in a specific AAF in a randomised controlled trial.^56^ However, there is evidence from basic and animal research that a high bifidogenic gastrointestinal microbiome may decrease the risk of infections and stimulate the development of a balanced immune system; possibly reducing inflammation and allergy.^57^ Furthermore, secondary outcomes in studies in presumed healthy and allergic infants reported a decrease in infectious disease and antibiotic prescriptions.^58^ Most guidelines do not recommend the routine use of pro‐, pre‐, syn‐ or postbiotics for the prevention or treatment of CMA. However, the recent DRACMA guidelines do recommend that ‘When choosing a formula with or without a probiotic for infants with IgE‐mediated CMA, we suggest either a formula without a probiotic or EHF (casein based) containing *Lacticaseibacillus Rhamnosus* (formerly *Lactobacillus rhamnosus*)’.^47^

### Complementary feeding in children with CMA

Early introduction of peanut to induce peanut oral tolerance became a primary prevention approach after the publication, in 2015, of the Learning Early about Peanut (LEAP) study.^59^ This was followed by the Enquiring About Tolerance (EAT)^60^ study, where only breastfed infants not at specific risk for developing food allergies were recruited. In this study, they included the early introduction of cow's milk, egg, wheat, peanut, sesame and fish. This study did not find a statistically significant reduction in the risk of developing CMA with the early introduction of cow's milk. However, they did confirm the previous results of the LEAP, that early introduction of peanut and egg was protective.^60^

While there are no specific recommendations for the introduction of complementary foods in children with an existing CMA, general infant feeding guidelines suggest introducing all allergenic foods when other complementary foods are introduced, around 6 months of age but not before 4 months of age.^61^, ^62^ There is no evidence that delaying the introduction of allergenic foods prevents the development of further food allergies in a child with existing CMA. The Dietary Guidelines for Americans 2020–2025^62^ highlights the nutritional value of allergenic foods in the infant diet stating, ‘It is *important* to introduce potentially allergenic foods along with other complementary foods’, and, ‘Protein foods, including meats, poultry, eggs, seafood, nuts, seeds, and soy products, are important sources of iron, zinc, protein, choline, and long chain polyunsaturated fatty acids’.

The role of diet diversity and specific dietary components in food has also recently become a target for the prevention of atopic disease. Diet diversity is the number of different foods, food groups, or food allergens that are included in the diet over a given period of time. The role that diet diversity plays in allergy prevention in early life, particularly food allergy prevention, has been recently described in the literature.^63^ It is postulated that ‘diet diversity’ can lead to changes in the microbiome, gut epithelial structure and immune profile. Despite the potential that diet diversity may significantly change the gut microbiome and, consequently, immune outcomes, there is a paucity of data about the role of dietary diversity specifically in the management of CMA. No studies have been conducted to determine the role of diet diversity in infancy and CMA outcomes. However, Maslin et al.^64^ showed that diet diversity in children excluding cow's milk in the first year of life was reduced compared to those consuming cow's milk. It may, therefore, be important to focus on increasing the diversity of the diet in children with diagnosed CMA.

## NUTRITIONAL CONSEQUENCES

The concern about growth, in particular longitudinal growth, was already highlighted by Isolauri et al.^65^ in 1998. Many studies have followed since then, confirming this as a possible consequence of CMA with around 10% suffering from stunting.^66^ Therefore, the majority of children with CMA do thrive along their growth centiles.^66^ In the past, poor growth was attributed to the elimination of cow's milk and its derivatives leading to a reduced intake of macro and micronutrients.^67^ While this may still be the case, poor growth has also been observed in children optimally managed through dietetic input.^65^, ^68^ This has highlighted other possible causes of poor growth, including ongoing inflammation (i.e., skin or gastrointestinal) and feeding difficulties, leading to a diet that was deficient in essential macro and micronutrients.^66^ Tumour necrosis factor α (TNF‐ α), interleukin 1β, and IL6 are pro‐inflammatory cytokines well known to impact longitudinal growth in children with inflammatory disease.^69^ D'Apolito et al.^70^ reported that several cytokines were elevated in children with CMA, including TNF‐α. However, to date, no study has been performed that can link ongoing inflammation, including cytokine levels, with poor height growth.

While impairment in growth is well recognised, Meyer et al.^71^ also observed cases of obesity in their international survey on the growth of children with food allergies. In that study, 8% of children had a BMI > 2 SD *z*‐scores, which is lower than the published prevalence of the general population by the World Health Organisation (2018) (18% of 5–19‐year‐olds are overweight or obese), but it highlights an important shift in children with food allergies which healthcare professionals need to consider in their management of CMA (https://www.who.int/news-room/fact-sheets/detail/obesity-and-overweight).

Feeding difficulties have been found to be associated with poor growth,^72^ and while they are commonly reported in children with food allergies,^73^, ^74^ there are no published studies showing an association between CMA, feeding difficulties and growth. However, there are some studies reporting an association between eosinophilic oesophagitis, feeding difficulties and poor growth.^74^, ^75^ Data overall is very limited, therefore more research focusing on the interaction between feeding difficulties, food allergy (CMA) and growth is required.

It is known that children with CMA are at a higher risk of micronutrient deficiencies. The data has very much focused in the past on calcium and vitamin D, but children with food allergies are at risk of multiple vitamin/mineral deficiencies.^66^, ^76^, ^77^ Standard dietetic practice, supported by guidelines, has always considered the supplementation of calcium and vitamin D, where required. Interestingly, a study from 2014 found low bone mineral density in young adults with IgE‐mediated CMA, which correlated with lower calcium, but not vitamin D, intake, and only improved through cow's milk desensitisation.^51^ The authors highlighted the importance of considering the co‐factors in the bioavailability of calcium, including the format, co‐nutrients (i.e., phosphate) and possibly interactions with medications (i.e., proton pump inhibitors).^78^

The most notable change in terms of micronutrient focus in children with CMA has come from the data published by Thomassen et al.^79^ in Norway, which highlighted that 58% of primarily breastfed infants were deficient in iodine. Iodine deficiency has been linked to impaired neurological development, with negative effects on child growth and development.^80^ Since this publication, extra attention has been paid to considering iodine sources when providing dietary advice, while more plant‐based alternatives are now also fortified with iodine.

With the increase in plant‐based nutrition, also in children there is an increased awareness on the nutritional complexities of a child consuming a plant‐based diet and having the diagnosis of CMA.^81^ Concerns have been highlighted about the ultra‐processed nature of plant‐based beverages, but they can also be a good source of calcium (and other micronutrients). It is important that healthcare professionals are optimally educated to provide individualised advice to maintain growth and prevent deficiencies and have a balanced view of the concerns of ultra‐processed foods, which is discussed in further detail by an EAACI Task Force report (reference to be added after release next week).

## NOVEL APPROACHES TO THE MANAGEMENT CMA

### Oral immunotherapy (OIT) to cow's milk

OIT is increasingly being considered as a therapeutic strategy to desensitise individuals with CMA, particularly those with persistent IgE‐mediated CMA. A systematic review by the DRACMA (guideline) group^82^ identified 2147 unique published records in the last decade, including 13 randomised trials and 109 observational studies discussing the use of cow's milk OIT. The group concluded that there was moderate certainty that OIT, with unheated cow's milk, in patients with IgE‐mediated CMA is associated with an increased likelihood of being able to consume milk, but found no specific volume that was universally tolerated between studies. However, they also acknowledged the increased risk of adverse effects. The DRACMA systematic review on OIT for patients with CMA, provides further details on the variation in protocol and outcomes, including sustained unresponsiveness.^82^

Despite the large number of papers published, the role of the dietitian in OIT is still unclear. Dietitians involved in OIT are mainly based at a handful of allergy specialist research centres. Groetch et al.^83^, ^84^ summarised the role of the dietitian in recent papers.

Dietitians play an important patient education role which includes measuring and preparing the OIT dose, integrating the dose into their daily diet, and transitioning them to food equivalents to ensure that intake of the target dose is achieved. The dietitian has a broader role in the OIT approach, which includes assessing dietary intake and assessment of nutritional status.

### Milk ladder for IgE‐mediated allergy

A major advancement in the last 10 years has been the data on tolerance to baked milk (BM) in those with CMA. Many children are tolerant to BM from onset or develop tolerance to BM prior to becoming tolerant to non‐BM. It is, however, unclear if regular consumption of BM products is safe, and whether it leads to tolerance development. A milk ladder is a stepwise progression from extensively heated to less heated foods. Heating decreases the allergenicity of food proteins in milk by destroying confirmational epitopes, so that the immune system has reduced ability to recognise them,^85^ although heating has minimal effect on linear epitopes.^85^ Thus, it is assumed that advancing from extensively heated to less heated foods offers a progression from a less‐allergenic to a more‐allergenic form of the food protein. Food ladders also consider the amount of allergenic protein in each step of the ladder, which progressively increases as you climb up the rungs of the ladder The first published ladder was created in 2013 for non‐IgE‐mediated CM allergy in the United Kingdom by Venter et al.^86^ It initially contained 12‐steps focusing on common British foods. In 2017 Venter and colleagues updated this ladder to a shortened version, which was more internationally focused and complied with World Health Organizations salt and sugar recommendations.^4^ This ladder has been widely adopted for non‐IgE‐mediated CM allergy.^87^ Although initially created for non‐IgE‐mediated allergies, ladders are also being used by many healthcare professionals for IgE‐mediated allergies, especially to egg and CM.^88^ In one survey they found that as many as 60% of healthcare professionals were using CM ladders for IgE‐mediated allergies.^87^ In a rostrum publication by Venter et al.,^89^ reviewing the current scientific basis for food ladders, their benefits and risks, and the recommendations for the future, they reported that the potential benefits of using a ladder approach for IgE‐mediated food allergy include (1) hastening of resolution of a food allergy,^90^ (2) increased diet diversity,^91^ (3) less healthcare utilisation, (4) decreased cost and (5) decreased patient burden.^89^ This rostrum also recommended standardisation of food ladders considering the allergenic protein content and cooking instructions for recipes, the nutrition and health value of foods and acceptance of the food by paediatric patients, as well as consideration for local/cultural eating habits. However, despite these benefits, there is limited evidence demonstrating induction of tolerance through ladders. More recently a group in Ireland used the MAP ladder for CM introduction in children with IgE‐mediated CMA (mean SPT 5.96 mm and specific IgE 11.3 kUA/L) starting at an average age of 7.3 months, following a negative supervised challenge of ED 05 (0.5 mg CM protein).^92^ They reported no severe adverse events when following the milk ladder at home, and 64% of the children following this approach were fully tolerant to CM by 12 months post randomisation; compared to just 37% of those using the standard diet avoidance approach. However, this needs to be approached with caution until more data is available, as near‐fatal or fatal reactions to milk, especially in individuals with asthma have occurred. Therefore, using ladders or other types of baked food approaches (in the home) is not without risks and guidance from a physician is essential.^93^

### Introduction to BM

In 2008, Nowak‐Wegrzyn et al.^94^ demonstrated that the majority of children with CMA tolerated BM. There is debate as to whether baked forms of CM accelerate tolerance to unbaked forms,^91^, ^95^, ^96^, ^97^ as it is likely that those who are BM tolerant have a more transient allergic phenotype compared to those with a more persistent phenotype, who do not tolerate baked forms. Nonetheless, the inclusion of BM results in a more liberalised diet and reduces the burden of avoidance. Furthermore, in those tolerating BM, inclusion in the diet may accelerate the development of unbaked CM tolerance compared with strict avoidance.^96^, ^98^

BM introduction in those with IgE‐mediated CM allergy was historically performed under physician supervision as an OFC, using a standardised recipe. These recipes (previously published by Bird et al.^99^) included approximately 1.33 g BM protein and were baked in the oven in a grain matrix at 350 degrees Fahrenheit for about 30 min. The recipes were developed with a high concentration of CM protein, higher than a typical baked‐good recipe. The benefit of using such a standardised recipe is that it informs the degree of tolerance for continued ingestion of BM ingredients, allowing most commercial baked goods containing BM protein, as the amount of BM ingredient is likely to be less than what was tolerated in the BM OFC.

Many recipes have been published, but there are still unanswered questions, such as how to proceed with evaluating tolerance to BM and how to provide education after tolerance has been achieved (Table 3).^100^

**Table 3:** Common approaches and unanswered questions regarding baked milk education (adjusted with permission from Groetch and Venter^100^).

Common approach	Common approach	Advise
Allow	Avoid	Caution
Ratio of ingredients in a bake product (e.g., muffin, cookie, cracker, or roll) is dependent on volume tolerated in an oral food challenge: Common published recipes allow 1.33 g baked milk protein per serving. Based on this amount, ratio should be no more than 1 cup of milk per 1 cup of flour with a yield of six servings unless the patient has tolerated a higher dose on oral food challenge.	More milk than what was tolerated on oral food challenge Dishes such as macaroni and cheese, lasagne, pizza are not baked goods and unless tolerated or passed with an oral food challenge, they should be avoided.	It is unknown if less matrix or more milk will be tolerated but may be on an individualised basis.
Store‐bought baked milk: Commercially baked products with milk ingredients listed as the third ingredient or further down the list of ingredients. This approach has been used successfully in multiple clinical trials. It is unknown if other products will be tolerated. Serving sizes are specified on the nutrition facts label.	Any commercial baked item with milk as the first or second ingredient. Any unbaked milk ingredient.	Ensure the ingredient is a *baked* ingredient. A cheese‐flavoured cracker may have the flavouring topically applied after the cracker is baked. Cakes or cookies may have unbaked ingredients in icing or frosting.
Cooking method/doneness. All baked milk products must be baked in the oven and must be cooked throughout to a dry‐crumb texture. A full‐size muffin would typically be baked at 350 degrees for 30 min, as in common published baked milk recipes.	Any item that is not a baked‐good (e.g., lasagne or macaroni and cheese) or any item that is cooked but not baked like pudding, custard, French toast, or heated milk. Milk chocolate chips that will melt during baking but not ‘bake’. Items that are not baked throughout or are wet, gummy or soggy in the middle. Avoid any unheated milk ingredients.	Smaller muffins/cupcakes/cookies will bake for around 15 min. Ensure they are baked thoroughly. It is unknown if less baking time will change allergenicity.

An individualised approach is appropriate if the patient has been evaluated for the degree of tolerance. For instance, Miceli Sopo et al.^101^ evaluated the need for a wheat matrix in those BM tolerant and found that a wheat matrix was required for some patients, but not all. The wheat matrix has been shown to be important as the interaction between proteins and carbohydrates/fats does impact the allergenicity of CM protein. Some, but not all, patients tolerant to BM also appear to be tolerant to (oven) baked cheese, for instance on pizza.^98^ Once a patient has a negative OFC to BM, education should be provided on foods that are allowed. The amount and type of BM product allowed should reflect the amount tolerated, the cooking method used, and the matrix used during the OFC.

BM has been explored as an approach to oral desensitisation using incremental amounts of BM in children not tolerant to a full dose.^102^, ^103^, ^104^ In a study by Dantzer et al.,^103^ they enroled participants who were not BM tolerant, based on DBPCFC, they then randomised participants to either placebo or BM OIT. After 12 months of OIT, 11 of 15 (73%) in the BM group tolerated the maximum cumulative dose of 4044 mg of BM protein, compared to 0 of 15 in the placebo group (*p* < 0.0001). Although the approach appeared safer than OIT to unbaked milk, reactions were common, including four dosing‐related reactions that required epinephrine. The long‐term risks and benefits of BM OIT, therefore, require more research.

## CONCLUSION

CMA remains one of the most common and complex paediatric food allergies. In the last decade, little has changed in terms of IgE‐mediated incidence, but more focus appears to be paid to non‐IgE‐mediated CMA, particularly in some Western countries. There has been significant progress in the last 10 years in relation to our understanding of existing supportive tests for IgE‐mediated CMA, including the advancement of cut‐off values in aiding the diagnosis along with newer tests such as BAT which have shown some promise. Meanwhile, no advancement has been made in terms of tests for non‐IgE‐mediated CMA and controversy still exists around symptoms, which overlap with other paediatric conditions, such as disorders of gut–brain interaction. Data on the natural history of CMA suggests a lower resolution than previously thought in IgE‐mediated CMA, which might suggest a change in phenotype although it could also be the result of reporting bias (coming from mainly specialist centres).

In terms of the evolution of dietary management of CMA this appears to have become more active. Breastmilk remains the gold standard for all infants. However, we now see new infant formula options on the market, including plant‐based hypoallergenic formula and milk alternatives, many of which are fortified. The addition of pro, pre and synbiotics remain controversial in terms of additional benefits, although they are considered safe to use, more research and guidance on routine use is required. Tolerance induction through modulation of the microbiome and diet diversity during complementary feeding has become a target for the prevention of atopic disease. This has been a major advancement in the last 10 years or so. While poor growth and feeding difficulties remain a concern in children with CMA, increased rates of obesity are now also commonly reported. Furthermore, micronutrient deficiencies, although long recognised as an issue for these children, have also come to the forefront in the last decade and have gone beyond calcium and vitamin D. Finally, novel approaches, including OIT, use of milk ladders and earlier consideration of BM, have become more popular in the last decade. However, the long‐term risks and benefits of these novel approaches require further research.

## AUTHOR CONTRIBUTIONS

All authors have equally contributed to this publication.

## CONFLICTS OF INTEREST STATEMENT

Dr. Rosan Meyer reports grants with Danona/Nutricia, honoraria from Reckitt Benckiser, Nestle Nutrition Institute, Danone, Abbott Nutrition and consultancy fees from Else Nutrition and CoMISS supported by Nestle Nutrition. Marion Groetch receives royalties from UpToDate and Academy of Nutrition and Dietetics and consulting fees from Food Allergy Research Education; serves on the Medical Advisory Board of IFPIES, as a Senior Advisor to FARE, as a Health Sciences Advisor for APFED; on the editorial board of Journal of Food Allergy; and has no commercial interests to disclose. Dr. Alexandra Santos reports grants from the Medical Research Council (MR/M008517/1; MC/PC/18052; MR/T032081/1), Food Allergy Research and Education (FARE), the Immune Tolerance Network/National Institute of Allergy and Infectious Diseases (NIAID, NIH), Asthma UK (AUK‐BC‐2015‐01), BBSRC, Rosetrees Trust and the NIHR through the Biomedical Research Centre (BRC) award to Guy's and St Thomas' NHS Foundation Trust, during the conduct of the study; personal fees from Thermo Scientific, Nestle, Novartis, Allergy Therapeutics, IgGenix as well as research support from Buhlmann and Thermo Fisher Scientific through a collaboration agreement with King's College London. Dr. Carina Venter reports grants from Reckitt Benckiser and personal fees from Reckitt Benckiser, Nestle Nutrition Institute, Danone, Abbott Nutrition, and Else Nutrition, outside the submitted work.

### PEER REVIEW

The peer review history for this article is available at https://www.webofscience.com/api/gateway/wos/peer-review/10.1111/jhn.13391.

## Data Availability

The data that support the findings will be available in DRACMA at https://www.worldallergyorganizationjournal.org/dracma-series following an embargo from the date of publication to allow for the commercialisation of research findings.
